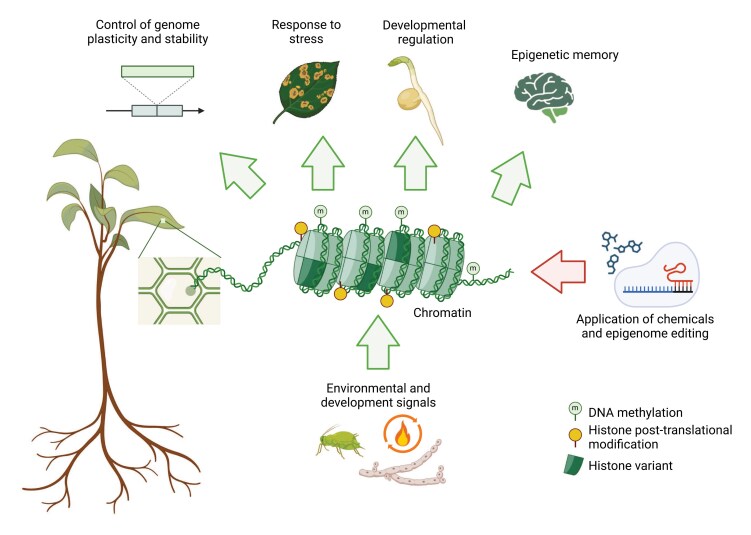# Plant epigenetics: controlling genome expression to integrate developmental and environmental cues

**DOI:** 10.1093/jxb/eraf134

**Published:** 2025-06-17

**Authors:** Marco Catoni, Tamara Lechon Gomez, Aline V Probst

**Affiliations:** School of Biosciences, University of Birmingham, Birmingham B15 2TT, UK; School of Biosciences, Cardiff University, Cardiff CF10 3AX, UK; iGReD, Université Clermont Auvergne, CNRS, INSERM, BP 38, 63001, Clermont-Ferrand, France

**Keywords:** DNA methylation, epigenetics, histone modifications, histone variants, Polycomb, transcriptional regulation

**Gene expression changes induced in response to developmental and environmental cues, as well as the maintenance and inheritance of these altered expression states, require epigenetic regulation. Epigenetic mechanisms comprise modifications at the chromatin level, including DNA methylation and alterations in nucleosome organization through the incorporation of specific histone variants and their post-translational modifications, as well as the involvement of various non-coding RNAs. Many of these processes and factors are evolutionarily conserved, while others are specific to photosynthetic organisms. This Special Issue is published in association with the EPIPLANT/SEB conference 2024 and the sessions dedicated to epigenetic plasticity and plant epigenetics at the SEB Prague 2024 conference, which brought together researchers working in model and crop species. It comprises reviews and original articles discussing insights into the epigenetic mechanisms in plants and other photosynthetic organisms, as well as avenues to improve their adaptability to the changing environment.**

The organization of the nuclear DNA into chromatin plays an important role in controlling the expression of the genome by establishing chromatin states that are either permissive or repressive for transcription. The basic subunit of chromatin is the nucleosome (Box 1), in which ~146 bp of DNA wrap around an octamer of the core histone proteins, H3, H4, H2A, and H2B. At certain loci and specifically at repetitive heterochromatic regions where it promotes higher order folding, the nucleosome can be bound by the linker histone H1 at the level of its dyad (Rutowicz *et al.*, 2019). Long chains of nucleosomes then organize into higher order structures, including chromatin loops, topologically associating domains, and chromosome territories (Doğan and Liu, 2018). Therefore, all cellular processes that operate on DNA, including transcription, take place in a chromatin environment. The nucleosome is generally considered to be an intrinsic barrier to transcription. To facilitate transcription elongation, the RNA polymerase II machinery associates with several transcript elongation factors (TEFs), including histone chaperones and chromatin remodelling complexes that modulate chromatin accessibility (reviewed in Grasser, 2025). To facilitate or repress transcriptional activity, chromatin organization can be modified, for example, by methylation of the DNA molecule itself, by incorporation of different variants of the core or linker histones that can directly affect nucleosome stability, or by post-translational modifications of these histone proteins that are read and interpreted by specific reader proteins. Together, these chromatin modifications act as ‘structural adaptations of chromosomal regions so as to register, signal or perpetuate altered activity states’, which are defined as epigenetic modifications (Bird, 2007).

Methylation of the DNA molecule is the most studied epigenetic modification and occurs in plants mainly on cytosines in the CG, CHG, and CHH context (H=A, C, or T). DNA methylation is set and maintained by several DNA methyltransferases, among which DNA METHYLTRANSFERASE 1 (MET1) catalyses the maintenance of CG methylation, which is the most methylated context occurring in plant genomes. Deletion of *MET1* results in generation of many stable epialleles in the model *Arabidopsis thaliana* (Mathieu *et al.*, 2007; Reinders *et al.*, 2009; Catoni and Cortijo, 2018), suggesting that *met1* mutations could also be used to generate epigenetic variation in crop plants. However, obtaining a full MET1 knockout mutant has been proven challenging to achieve in species other than Arabidopsis, possibly due to the more important role of epigenetic regulation in plants with a larger transposon-rich genome. In this context, Burrows *et al.* (2025) have exploited the partial redundancy between *MET1* homoeologues in polyploid wheat to generate mutants with different MET1 dosage, and characterized changes in transcription and DNA methylation as a function of functional copies remaining. Interestingly, they also observed phenotypic trait variation associated with DNA demethylation, which would suggest the presence of epialleles in wheat.

Introducing DNA methyltransferase mutations is not the only means to study the functional significance of DNA methylation, and the use of non-methylable cytidine analogues has been common laboratory practice to reduce DNA methylation levels. The use and molecular mechanisms of action of these cytidine analogues are discussed in a review by Dvořák Tomaštíková and Pecinka (2025). Cytidine analogues, which are incorporated into DNA, probably act by covalently trapping the methyltransferases on DNA, thereby depleting active DNA methyltransferases. While the use of these chemicals represents a quick, easily applicable, and straightforward system to reduce DNA methylation in plant genomes, the plethora of their effects is not entirely understood, and also include changes of a non-epigenetic nature caused by DNA damage. Emphasizing possible ‘side effects’ is important for the interpretation of observed phenotypes in plants exposed to cytidine analogues, which might not be a direct consequence of epigenetic misregulation. Cytidine analogues are excellents tools to investigate epigenetic regulation in non-model plant species with limited access to genetic resources and mutant collections. While initially described to lead to the reactivation of silent genetic elements, cytidine analogues have later been applied to enhance transgene expression, to induce somaclonal variation, or to modify flowering time (Dvořák Tomaštíková and Pecinka, 2025). Examples of a role for DNA methylation in controlling flowering in different species are reported in this issue. Yang *et al*. (2025) describe a global increase in CHH methylation associated with long-day growth conditions in the forage crop orchardgrass. With the use of cytidine analogues, the authors managed to induce late flowering under long-day conditions, suggesting a functional role for DNA methylation in the control of flowering in this species. Yuan *et al.* (2025) studied the DNA methylation patterns in *Angelica sinensis*, a Chinese herbal plant, comparing methylation profiles in plants before and after bolting, linking DNA methylation to the biosynthesis of lignin and other phenylpropanoid compounds. Garro *et al.* (2025) further identified a role for DNA methylation in regulating photomorphogenesis in Arabidopsis in response to warm temperatures. Together these studies provide additional examples of the importance of DNA methylation in gene regulation and developmental processes.

Epigenetic modifications can also be mitotically inherited and even transmitted to subsequent generations, providing transgenerational memory to the offspring. Therefore, studying epigenetic regulation of meristematic cells, which constitute the plant germline, is useful to understand how transgenerational information could be transmitted. Despite the inherent difficulties in investigating the small meristem tissue in plants, several studies have provided insight into the role of epigenetic mechanisms, particularly DNA methylation, in the maintenance of the stem cell pool in the shoot apical meristem (SAM) and in SAM-related developmental processes, as reviewed in this issue by Yang and Johannes (2025). The authors discuss the genetic evidence for a role for DNA methylation in SAM function obtained from DNA methylation mutants, as well as the observation that one of the central regulators of the SAM, the transcription factor WUSCHEL, directly interacts with ARGONAUTE 4, which recruits the RNA-directed DNA methylation machinery (RdDM) to the target promoters, thereby suppressing their expression (Zeng *et al.*, 2023). DNA methylation changes in the SAM can also occur in response to stress or during development, providing a mechanism to establish a form of stress memory (Yang and Johannes, 2025).

Bart and Wang (2025) further discuss the role of DNA methylation in plant development and the response to external stimuli. Indeed, mutants of DNA methyltransferases or plants carrying certain epialleles (loci that differ only in the epigenetic state and not in the DNA sequence) can show altered responses to abiotic and biotic stresses. Therefore, modifying DNA methylation may present a way to increase crop resistance. In recent years, a number of genome editing tools have been developed that rely on zinc finger nucleases or CRISPR/Cas9-coupled strategies associated with epigenetic modifiers to target chromatin modifications at specific genes. In contrast to simple knockouts or constitutive overexpression of epigenetic modifiers, these techniques may offer the possibility of fine-tuning gene expression in a stable or transient manner without introducing variation in the DNA sequence.

Finally, DNA methylation cannot be regarded in isolation as it is tightly linked with other chromatin modifications. This has, for example, been demonstrated by existing feedback loops such as the recognition of CHG methylation by the H3K9 histone methyltransferase KRYPTONITE/SUVH4 (KYP) and in turn the role of H3K9me2 methylation in the recruitment of CHROMOMETHYLASE3 (CMT3) (Jackson *et al.*, 2002; Johnson *et al.*, 2007). Furthermore, critical steps of cellular life such as DNA replication, which is tightly coupled to the replication of chromatin modifications, have demonstrated the close link between DNA methylation maintenance, nucleosome assembly, and the replication-coupled methylation and (de)acetylation of the newly incorporated histones (Groth *et al.*, 2007). In this Special Issue, Edera and Quadrana (2025) discuss the link between DNA methylation and the deposition of core histone variants and linker histones. The linker histone H1 has been shown to prevent methyltransferases from accessing DNA, which therefore requires an ATP-dependent chromatin remodeller called DECREASE IN DNA METHYLATION 1 (DDM1) to methylate DNA (Zemach *et al.*, 2013). Recent genetic and structural data show that DDM1 promotes the incorporation of the replicative H3 histone variant H3.1 (Lee *et al.*, 2023) and the heterochromatin-enriched H2A variant H2A.W (Osakabe *et al.*, 2021), hinting at a close interplay between DNA methylation, the incorporation of core histone variants, and linker histones, processes in which DDM1 acts as a central player.

The development of multicellular organisms requires the activation and repression of sets of genes and the stable maintenance of gene expression states over multiple cell divisions. The evolutionarily highly conserved Polycomb/Trithorax system (Vijayanathan *et al.*, 2022) has been implicated in the regulation of developmental as well as stress-responsive genes. Polycomb repressive complexes (PRCs) 1 and 2, comprising histone H2A ubiquitination and H3K27 methyltransferase activity, respectively, are required for the stable repression of developmental genes. In this Special Issue, Baldwin *et al.* (2025) have examined the genomic enrichment of H3K27me3 in ripe strawberries after harvest and cold storage, and identified a set of genes repressed by the Polycomb mark including cold-responsive genes linked to colour and aroma production. In another study, Prasad *et al*. (2025) studied transcriptome changes and the distribution of both H3K4me3 and H3K27me3 in a genome-wide manner in rapeseed plants exposed to water-limiting stress induced by polyethylene glycol (PEG) treatment, and identified a correlation between these epigenetic marks and expression of genes involved in biosynthesis of osmoprotectant compounds. While global gene repression is reflected by the presence of specific histone modifications, dynamic changes in gene expression do not always correlate with changes in the histone modification profile (Liu *et al.*, 2014; Holder and Deal, 2024, Preprint). This may suggest that transcriptional activation can occur despite the presence of repressive marks and that changes in chromatin states may occur with a temporal lag or may rather reflect more permanent gene expression states. Genetic tools to induce gene expression changes at specific genes followed by temporal analysis of chromatin states, ideally even at the single-cell level [see reviews by Wang and Bart (2025) and Yang and Johannes (2025)], should help to further define the relationships between histone modifications and gene expression.

While we have gained mechanistic insights into the function of the PRCs and their recruitment to chromatin, how signals perceived by the plant are translated into chromatin changes is still an open question. Dong *et al.* (2025) discuss the role of the kinase Target of Rapamycin (TOR), a central regulator of cellular metabolism, in signalling chromatin functions. TOR may act directly via binding of chromatin modifiers or more indirectly by regulating the translation of chromatin proteins. For example, TOR promotes the relocalization of FERTILIZATION-INDEPENDENT-ENDOSPERM (FIE), an essential component of PRC2, from the cytoplasm to the nucleus, providing a link between TOR signalling and Polycomb function (Ye *et al.*, 2022).

Large-scale changes in nucleosome composition and histone modifications, but also in higher order chromatin organization, have been implicated in developmental transitions such as seed development and germination, which require important and controlled changes in the gene expression programme. Tremblay and Qüesta (2025) review how histone modifications, histone variants, and DNA methylation as well as non-coding RNAs and higher order chromatin organization act to regulate the expression of genes critical for controlling seed maturation and germination and for integrating developmental cues. This is an example of the complex interplay that exists between the different layers of epigenetic information. Together, these different layers allow several stimuli to be signalled and perhaps buffered and integrated with pre-existing patterns of information that allow for rapid reprogramming of the gene expression programme and an optimal response. Further insight into this complex interplay and the causal relationships between epigenetic marks and gene expression will require the development of new technologies in plants. Such technologies should include approaches that provide information on dynamic processes, such as inducible systems and the specific targeting of chromatin modifiers to genes, or single-cell technology that combines microscopy and genomic studies to assess variation between cells of a tissue and between individual plants.

Finally, epigenetic regulation plays an important role in controlling the activity of mobile DNA sequences, including transposable elements and viral-derived sequences that are integrated into the nuclear genome or found as extrachromosomal DNA molecules (ecDNAs) (Zhang *et al.*, 2023). In this Special Issue, Emmerson and Catoni (2025) describe such mobile DNA sequences and discuss their role in genome plasticity and as potential evolutionary drivers. What emerges is a very dynamic role for mobile elements in controlling genomic variation, leading to genetic rearrangements that facilitate DNA exchange and promote the emergence of new genes and functions.

In conclusion, this Special Issue captures well several aspects of the current studies occurring in the field of plant epigenetics, including different mechanisms such as developmental regulation, response to environment, and transgenerational memory, mediated by different pathways such as DNA methylation, chromatin modifications, and genome plasticity. The articles published here point to a significant effort to translate epigenetic research from *A. thaliana* to crops and non-model species, a process facilitated by a better understanding and optimization of existing tools (e.g. use of cytidine analogues) and by the generation of new approaches of epigenome editing. Plants have contributed significantly to the discovery of epigenetic regulation and are likely to continue to be a cornerstone for advancing our understanding of epigenetics. Moreover, the ongoing exploration of plant epigenetics holds promise not only for basic science, but also for practical applications that can address global challenges in agriculture and food security.

Box 1. **The organization of genomic DNA into chromatin permits a dynamic regulation of the gene expression programme**

Environmental and developmental signals that need to translate into an altered gene expression programme may elicit changes at the level of chromatin organization including the methylation of cytosines in the DNA molecule, the incorporation of specific histone variants into the nucleosome, and the setting of histone post-translational modifications. Together these different layers of epigenetic information allow the control of genome plasticity (e.g. by controlling mobile genetic elements), and permit the establishment of transient or heritable gene expression states to orchestrate developmental programmes and stress responses. The development of tools including cytidine analogues or epigenome editing provides the opportunity to artificially interfere with epigenetic regulation and to investigate chromatin-based epigenetic mechanisms. Created in BioRender. Catoni, M. (2025) https://BioRender.com/d72j736.